# Water physicochemical properties shape the distribution of submerged macrophytes: implications for wetland restoration in Songnen Plain

**DOI:** 10.3389/fpls.2025.1716202

**Published:** 2025-12-05

**Authors:** Pei Wang, Yinian Long, Long Wang, Rui Lu, Enrong Xiao, Zhenbin Wu

**Affiliations:** 1 State Key Laboratory of Breeding Biotechnology and Sustainable Aquaculture, Wuhan, China; 2 Institute of Hydrobiology, Chinese Academy of Sciences, Wuhan, China; 3 University of Chinese Academy of Sciences, Beijing, China

**Keywords:** submerged macrophyte, water physicochemical properties, biomass distribution, HCO_3_^-^ utilization, wetland restoration

## Abstract

Submerged macrophytes play a pivotal role in maintaining the clear-water state and enhancing biodiversity in wetland ecosystems. However, the environmental mechanisms underlying their assemblage and biomass patterns remain poorly resolved in continental alkaline wetlands. Here, we conducted a 27 hydrochemical parameters analysis and dissected its relations with the distribution of submerged macrophytes in Momoge wetland of the Songnen Plain, Northeastern China. The results revealed that rock weathering and evaporation-crystallization processes jointly regulated the baseline alkalinity and salinity of the water, thereby determining 62.5% species of submerged macrophytes capable of utilizing HCO_3_^-^ as an alternative carbon source. In contrast, nutrient inputs and wind-induced resuspension caused fluctuations in physicochemical conditions between light (50 < TLI ≤ 60) and moderate (60 < TLI ≤ 70) eutrophic states, resulting in *Potamogeton pectinatus*, *Najas marina*, and *Chara* sp*iralis* thriving in nutrient-rich, low-transparency waters, whereas *Utricularia aurea* and *Ceratophyllum demersum* favored clearer and less nutrient-enriched conditions. These findings highlight a two-tiered environmental control over submerged macrophytes in boreal wetlands, whereby geochemical processes shape species assemblages, and nutrient dynamics and physical disturbance drive biomass allocation. We propose a restoration strategy that combines species configuration and pilot selection, prioritizing HCO_3_^-^-utilizing pioneer species in degraded zones to gradually re-establish submerged macrophytes and ecosystem functions.

## 1 Introduction

Wetlands are vital ecosystems that provide numerous environmental services, including biodiversity conservation, water quality regulation, carbon sequestration, and flood control (Mitsch, 1993; Mitsch and Gosselink, 2000; Mitsch et al., 2015; Mio et al., 2024). As one of the most productive ecosystems on Earth, wetlands support a diverse range of plant and animal species and are considered biodiversity hotspots (Berde et al., 2022; Sharma and Naik, 2024). Submerged macrophytes, grow beneath the water surface, play a crucial role in wetland ecosystems by regulating water quality, stabilizing sediments, and enhancing biodiversity (Reid et al., 2019; Jeppesen et al., 2012; Chao et al., 2024). By occupying unique ecological niches, submerged macrophytes contribute to the transition from turbid to clear-water states (Ibáñez and Peñuelas, 2019; Zhang et al., 2022), thereby improving the overall health and functioning of aquatic ecosystems (Han et al., 2024; Jeppesen et al., 2012). Given their ecological importance, the decline in submerged macrophyte communities represents a significant threat to wetland health, calling for effective restoration strategies (Rodrigo, 2021).

Despite the crucial role submerged macrophytes play in wetland ecosystems, they face increasing threats from anthropogenic activities (Zhang et al., 2024), such as land-use change (Bomfim et al., 2025), nutrient loading (Dong et al., 2022), groundwater overexploitation (Puche et al., 2024), and climate change (Lind et al., 2022). These stressors, particularly nutrient enrichment and water quality degradation, have led to the decline of submerged macrophytes in many wetlands, disrupting ecosystem services and biodiversity (Liu et al., 2020; Kumar et al., 2023). The ongoing degradation of submerged macrophytes highlights the need for a comprehensive understanding of the factors that influence their distribution and abundance, which is critical for developing targeted wetland restoration strategies. Submerged macrophytes interact closely with their aquatic environment (Jeppesen et al., 2012), and their growth (Wang et al., 2020), species composition (Liu et al., 2020), and biomass distribution (Wang et al., 2015) are strongly influenced by hydrochemical properties such as pH, alkalinity, salinity, and nutrient concentrations. In particular, water chemistry plays a central role in shaping the structure of submerged macrophytes (Bornette and Puijalon, 2011; Ferreira et al., 2018). In regions with high alkalinity and salinity, submerged macrophytes often rely on HCO_3_^-^ as an alternative carbon source for photosynthesis (Iversen et al., 2019; Wang et al., 2017; Wang et al., 2024), which enables them to thrive in these environments (Maberly et al., 2015; Szabó et al., 2025). Furthermore, nutrient loading—especially nitrogen and phosphorus—can drive shifts in submerged macrophytes, favoring species that are more tolerant of eutrophic conditions (Ibáñez and Peñuelas, 2019). Understanding the complex interactions between inorganic carbon availability, nutrient loading, and hydrochemical factors is essential for predicting how submerged macrophyte communities will respond to environmental changes.

Although there has been significant research on the role of hydrochemical properties in shaping submerged macrophytes (Søndergaard et al., 2010, 2022; Szoszkiewicz et al., 2025), few studies have examined the specific interactions between water chemistry and submerged macrophytes in boreal wetland ecosystems, where species composition and water quality are highly sensitive to both natural processes and anthropogenic pressures (García Molinos et al., 2016; Xia et al., 2022). Boreal wetlands, such as those distributed in the Songnen Plain, are particularly vulnerable to changes in water chemistry due to nutrient enrichment (Luo et al., 2025), land-use changes (Chen et al., 2018), and fluctuating water tables (Zhang et al., 2015). Despite this, comprehensive studies that assess the relationships between hydrochemical parameters and submerged macrophytes in these regions are limited. This study aims to fill this gap by investigating the interactions between hydrochemical properties and submerged macrophyte distribution in the Momoge wetland of the Songnen Plain, Northeast China. By addressing the influence of hydrochemical properties on submerged macrophytes, this study will contribute to a better understanding of the environmental factors that govern the distribution of submerged macrophytes. Furthermore, it will provide practical guidance for restoring submerged macrophytes in boreal wetlands, with the ultimate goal of enhancing biodiversity, improving water quality, and ensuring the long-term sustainability of these vital ecosystems in the Songnen Plain wetlands.

## 2 Methods

### 2.1 Study area

**Figure 1:**
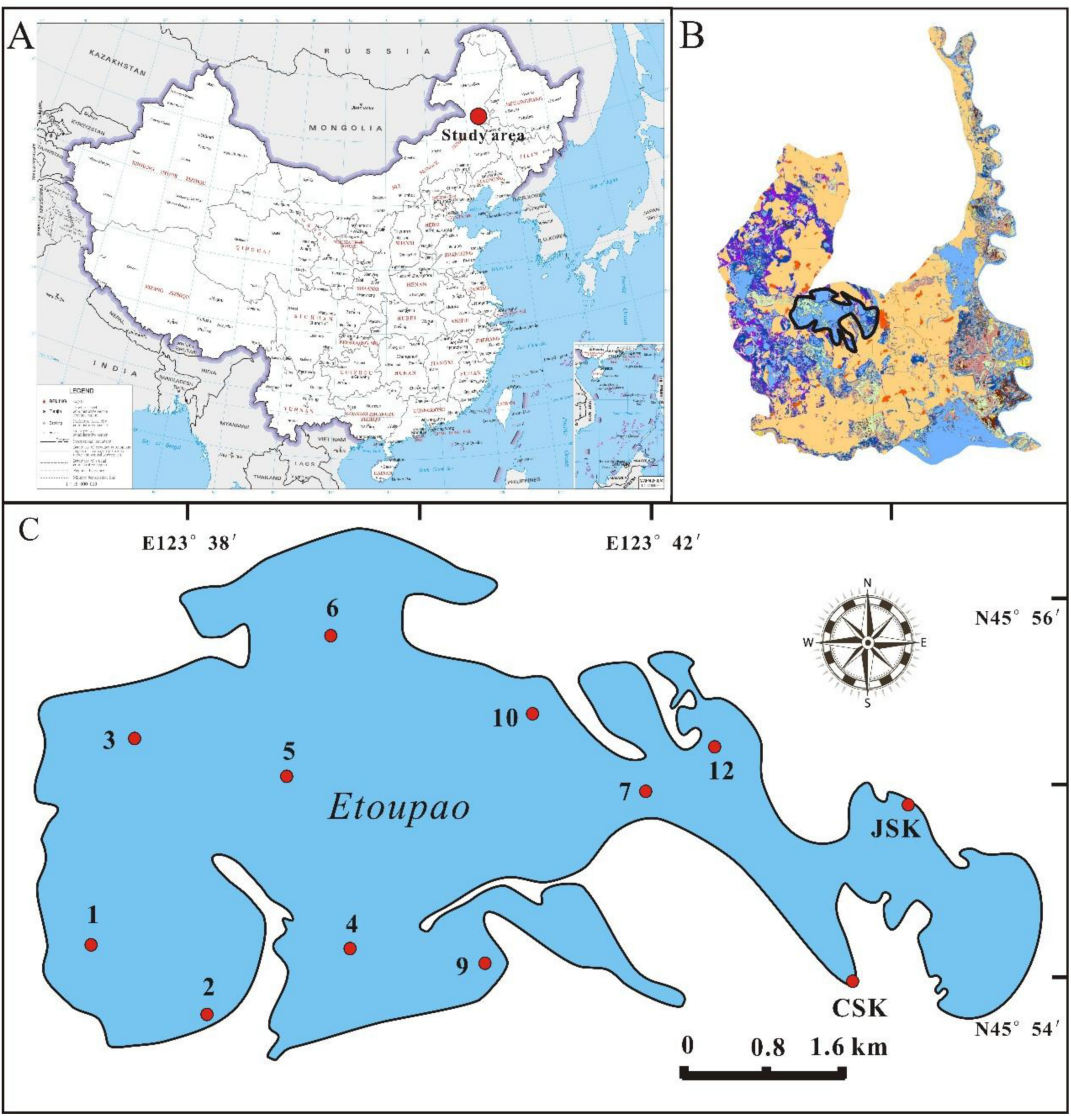
Situation of Momoge National Nature Reserve in China **(A)** and the study area in Momoge wetland circled in solid black line **(B)**, and the distribution of sampling sites in Etoupao **(C)**.

Momoge National Nature Reserve is located on the western margin of Songnen Plain, with the coordination of 45°12’25”N to 46°18’0”N, 123°27’0”E to 124°04’33”E and the average elevation is 142 m. The total area is 1.44×10^3^ km^2^, in which 80% consists of wetlands, such as lakes, rivers, and marshes. Etoupao which is the biggest one in Momoge wetland was chosen as the study area and the grid method (**Supplementary Figure S1**) was adopted to set up the twelve sampling sites distributed homogeneity (**Figure 1**). Typical temperate continental monsoon climate governs this region which has a frost-free period of 137 days, and the annual average temperature, solar radiation, precipitation, and evaporation are 4.2 °C, 5220 MJ m^-2^, 392 mm, and 1553 mm, respectively (Wei et al., 2019). In this nature reserve, approximately 60–000 residents live in and the land-use has been significantly changed for the purpose of raise yield (Jiang et al., 2007). It’s reported that the farmland and alkali-land increased by 168.9 km^2^ and 143.7 km^2^ in the twenty years (Li et al., 2018). Located in northeastern China where the arid fragile ecosystem, wetlands play an important role in regulating regional climate, increasing local rainfall, reducing sand-blown weather, elevating crop yield and protecting biodiversity etc., especially serve as a significant habitat and stopover site for the Siberian Crane (Jiang et al., 2016), which is the world’s most endangered crane species.

### 2.2 Field methods

Due to the water areas are frozen-up from late October to early May the following year including spring and winter (Huang et al., 2005), sampling surveys were conducted on September 6^th^-7^th^, 2017 (referred to as “Autumn”) and July 26^th^-27^th^, 2018 (referred to as “Summer”), and the water depth ranged from 0.30 to 1.50 m in the wetland, with a mean value of 0.98 m. At each site, physicochemical parameters of surface water samples were divided into two parts, *in situ* measurement and laboratory analysis. Water temperature (T), electrical conductivity (Ec), pH, dissolved oxygen (DO), oxidation-reduction potential (ORP) and salinity (Sal) were recorded *in situ* using portable equipment Manta YSI (Eureka, USA). Water transparency (Trans) and depth were measured by Secchi plate and depth meter (Speedtech, Japan), respectively. Water samples were collected and stored in a portable icebox for laboratory analysis of major water elemental components (K^+^, Na^+^, Ca^2+^, Mg^2+^, SO_4_^2-^, Cl^-^, HCO_3_^-^ and CO_3_^2-^), followed by the determination of total nitrogen (TN), nitrate nitrogen (NO_3_-N), nitrite nitrogen (NO_2_-N), ammonium nitrogen (NH_4_-N), total phosphorus (TP), dissolved inorganic phosphorus (DIP), chemical oxygen demand (COD), chlorophyll a (Chla), alkalinity (Alk) and total suspended solid (TSS).

Submerged macrophyte surveys were carried out by the quadrat method (Wang et al., 2015). A criterion comprising as many species as possible within a certain area was obeyed after walking through the wetland to evaluate the structure, composition, and variability of submerged macrophytes (Klee et al., 2019). At each sampling site, three quadrats were randomly selected with the size of 1 m×1 m, and to lower down the destruction of submerged meadows, five sub-quadrats (0.2 m×0.2 m) which distributed in the four angles and center of the quadrat were designated to harvest all the submerged macrophytes (Wang et al., 2015). After washed and wiped out all the water, fresh weights were calculated by electronic scale according to different species. Consequently, fresh weights for different species, average biomass at each sampling site, and composition of each quadrat were recorded at all sampling sites.

### 2.3 Laboratory analysis

The water samples were analyzed for K^+^, Na^+^, Ca^2+^, and Mg^2+^ by Inductively Coupled Plasma-optical emission spectroscopy Optima 8000DV (PekinElmer, USA). The SO_4_^2-^ and Cl^-^ were measured by Advanced Compact Ion chromatography (Metrohm, Switzerland). HCO_3_^-^ and CO_3_^2-^ as well as Alk were titrated by sulphuric acid with a concentration of 0.025 mol L^-1^ (Wang et al., 2020). In accordance with the 2002 “Water and Wastewater Monitoring and Analysis Methods” released by the Ministry of Environmental Protection of the People’s Republic of China (Lu et al., 2016), TN, NO_3_-N, NO_2_-N, NH_4_-N, TP, and DIP were determined by alkaline potassium persulfate digestion-UV spectrophotometric method, spectrophotometric method phenol disulfonic acid, spectrophotometric method with sulphanilamide and coupling with N-(1-napththyl)-ethylenediamine, Nessler’s reagent spectrophotometric, and ammonium molybdate spectrophotometric method, respectively. COD, Chla and TSS were determined by the potassium dichromate method (Lu et al., 2016), spectrophotometric method (Richardson et al., 2002), and gravimetric method (Lu et al., 2016). The nutrient levels were classified as oligotrophic (0.0-1.0 mg L^-1^) and mesotrophic (1.1-5.0 mg L^-1^) for nitrate concentrations and eu-polytrophic (0.031-0.1 mg L^-1^) and polytrophic (>0.1 mg L^-1^) for total phosphorus concentrations (Li et al., 2007), and the trophic level index (TLI) was calculated according to the Chla, TP, TN, SD, and COD (Carlson, 1977; China National Environmental Monitoring Centre, 2001).

pH-drift analysis was performed to test the inorganic carbon utilization by submerged macrophytes in the process of photosynthesis (Maberly, 1996). Fresh apical shoots were chosen as materials and wash out the attachments, then rinsed twice in the test solution which comprised equimolar concentrations of NaHCO_3_ and KHCO_3_ with the concentration of 1.0 mmol L^-1^. Plant shoots with the length of 6 cm were transferred into 30 mL glass tubes with stopper where had injected 25 mL Na/KHCO_3_ solution with the concentration of 1.0 mmol L^-1^ and the residual space were to alleviate the oxygen tension in solution (Yin et al., 2017). The culture systems were incubated in artificial climate incubator (Yiheng, Shanghai) at 25 °C and 75 µmol photon m^-2^ s^-1^. After 24 h continuous incubation, the final pH (there was no further change) was determined by Manta YSI (Eureka, USA).

### 2.4 Data analyses

Firstly, the water type was analyzed and visualized by GW_Chart, a program for creating specialized graphs according to the concentrations of K^+^, Na^+^, Ca^2+^, Mg^2+^, HCO_3_^-^, CO_3_^2-^, SO_4_^2-^, Cl^-^ and TDS. To trace the origins of the major ions and their controlling factors in the water columns, the Gibbs model (Gibbs, 1970) and ion ratios diagrams (Lakshmanan et al., 2003) were generated by Origin 85, respectively. Multivariate statistical analysis of the water physicochemical parameters was executed by SPSS 18, and the heatmaps were generated by R with corrplot packages to cluster the water physicochemical characteristics. Secondly, the relative frequency (RF), relative biomass (RB), and dominance (D) of submerged macrophytes were calculated following the method by Wang et al. (2015). And surfer 9 was employed to simulate the biomass distribution in the wetland. Finally, the canonical correspondence analysis was performed to figure out relationships between water physicochemical properties and biomass distribution of submerged macrophytes by Canoco for Windows 4.5. In the processes, CorelDRAW X4 was used not only to draw the sampling distribution map but also to integrate several figures into a complex.

## 3 Results

### 3.1 Hydrochemical properties

**Figure 2:**
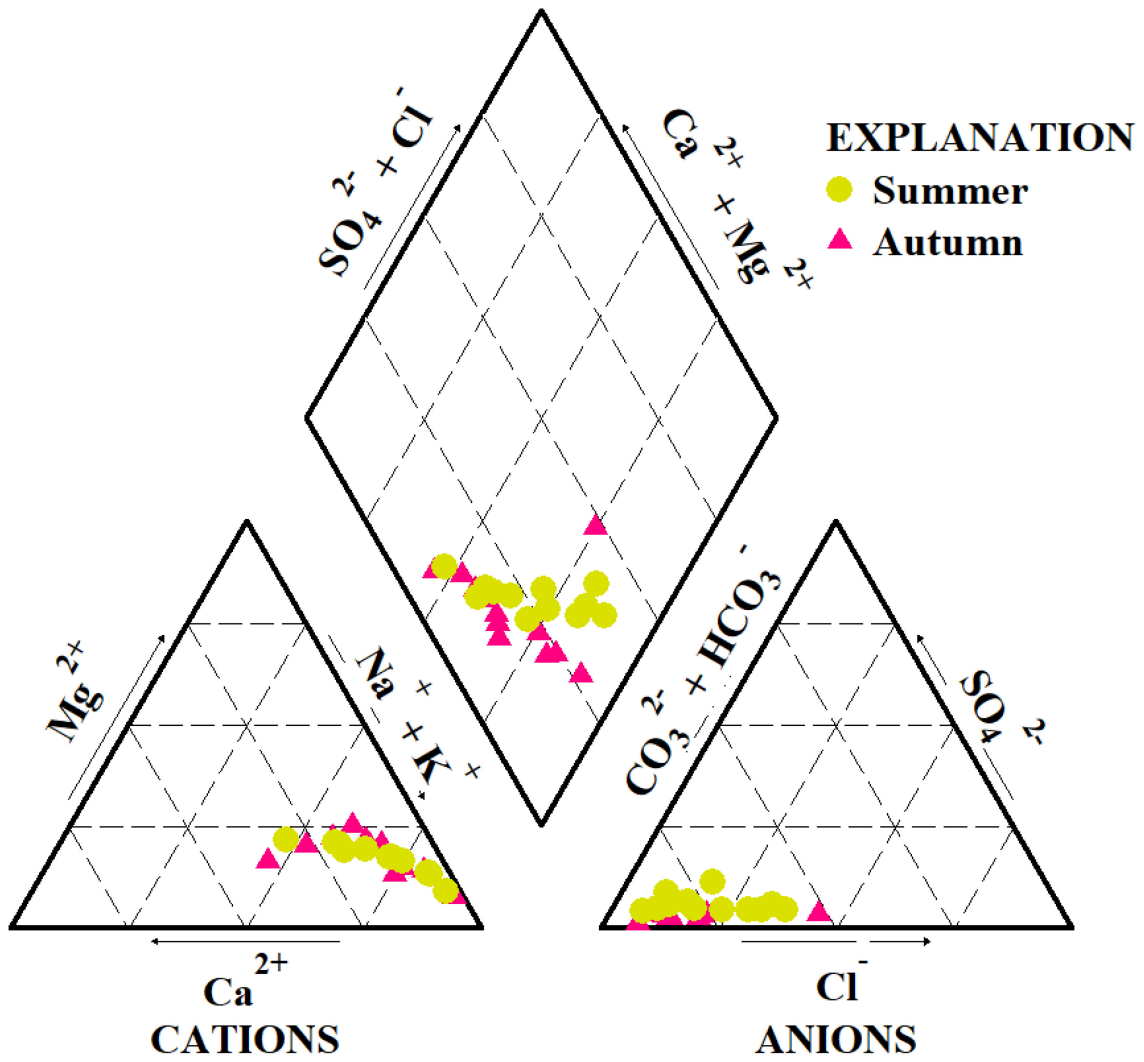
Piper diagram of water chemistry in the study area.

**Figure 3:**
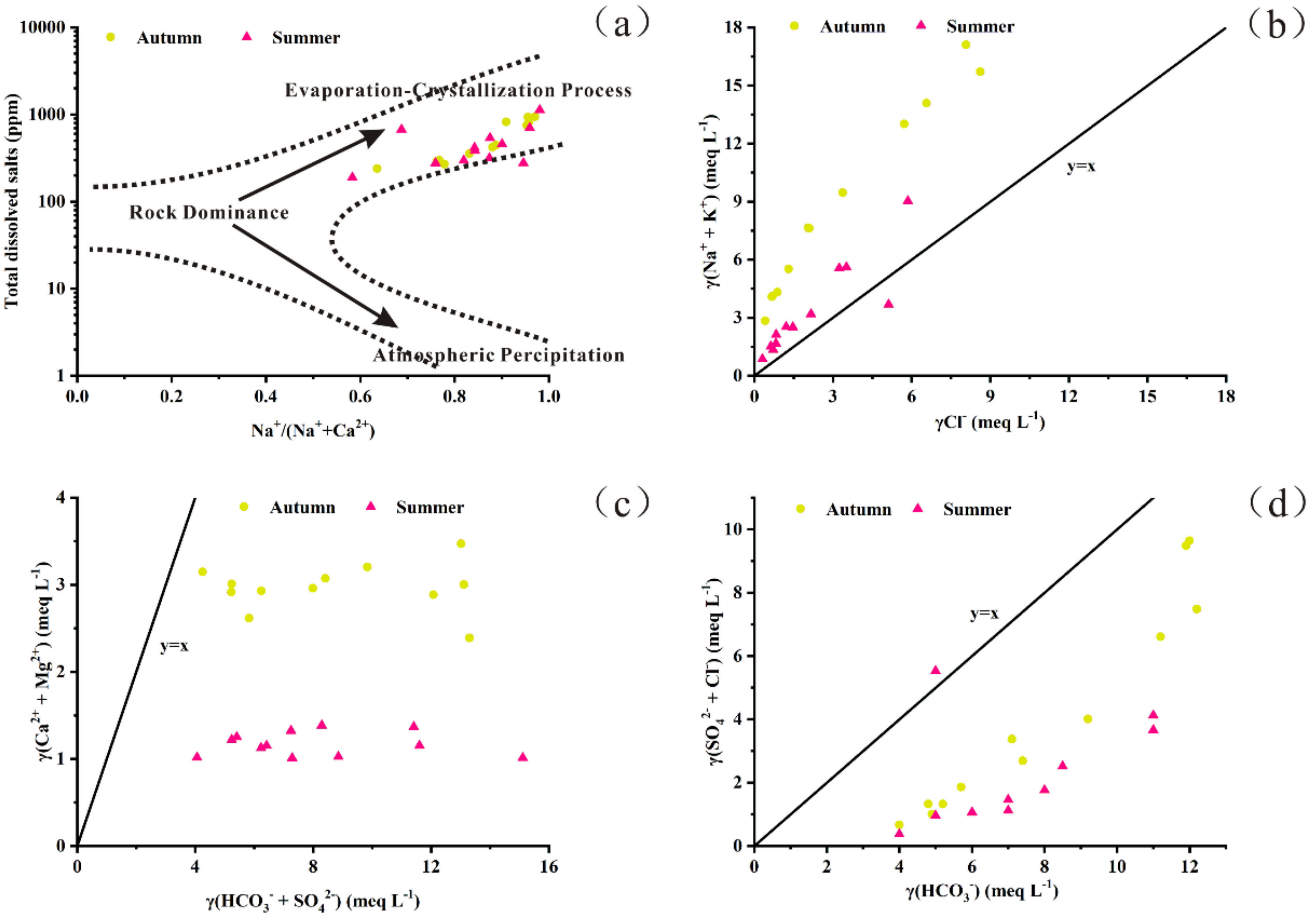
Relationships between the ratios of selected ions. **(a)** The diagram of Gibbs model. **(b)** The ratio between γ(Na^+^+K^+^) and γCl^-^. **(c)** The ratio between γ(Ca^2+^+Mg^2+^) and γ(HCO_3_^-^+SO_4_^2-^). **(d)** The ratio between γ(SO_4_^2-^+ Cl^-^) and γHCO_3_^-^. The y=x denotes the equilibrium line.

The abundance of cations followed the order Na^+^ > Mg²^+^ > Ca²^+^ > K^+^, while anions were dominated by HCO_3_^-^ > Cl^-^ > CO_3_²^-^ > SO_4_²^-^ in both sampling seasons. Overall, mean ion concentrations were higher in autumn than in summer (**Table 1**). Although the concentrations of major ions varied seasonally, the Piper diagram (**Figure 2**) indicated that the water consistently exhibited HCO_3_-type and Cl-type characteristics throughout the year. Ion ratio analyses further revealed distinct geochemical patterns. According to the Gibbs model, nearly all values clustered near 1 (**Figure 3a**). The ratios of γ(Na^+^ + K^+^)/γCl^-^ were primarily distributed above the 1:1 equilibrium line (**Figure 3b**), suggesting enrichment of sodium and potassium relative to chloride. In contrast, both γ(Ca²^+^ + Mg²^+^)/γ(HCO_3_^-^ + SO_4_²^-^) and γ(SO_4_²^-^ + Cl^-^)/γHCO_3_^-^ ratios were below the equilibrium line (**Figures 3c, d**), indicating that carbonate weathering and evaporative processes were dominant.

**Table 1:** Ranges of main ions and TDS in the water columns.

Types (mg L^-1^)	Autumn			Summer		
	Range	Mean	SD	Range	Mean	SD
Ca^2+^	11.77-36.74	22.56^a^	7.35	4.11-14.05	9.36^a^	3.17
Mg^2+^	15.60-31.69	22.08^a^	4.93	3.78-12.07	8.43^a^	2.19
Na^+^	64.17-389.40	199.76^b^	33.08	19.65-206.44	75.24^b^	54.16
K^+^	2.20-7.01	4.31^a^	1.40	0.37-2.46	1.29^a^	0.71
CO_3_^2-^	18.00-84.00	42.00^d^	5.12	0-150.00	32.50^d^	43.30
HCO_3_^-^	244.00-744.20	485.97^d^	193.48	244.00-854.00	470.21^d^	181.36
Cl^-^	14.66-306.02	119.76^d^	108.37	11.11-208.01	76.64^d^	66.15
SO_4_^2-^	12.17-67.43	35.94^c^	17.61	3.03-53.42	18.79^c^	12.67
TDS	239.80-944.00	549.44^a^	279.39	190.10-1131.00	472.47^a^	261.76

The significance levels represented by the letters a (p < 0.001), b (p < 0.01), c (p < 0.05), and d (p ≥ 0.05), based on the p-value.

**Figure 4:**
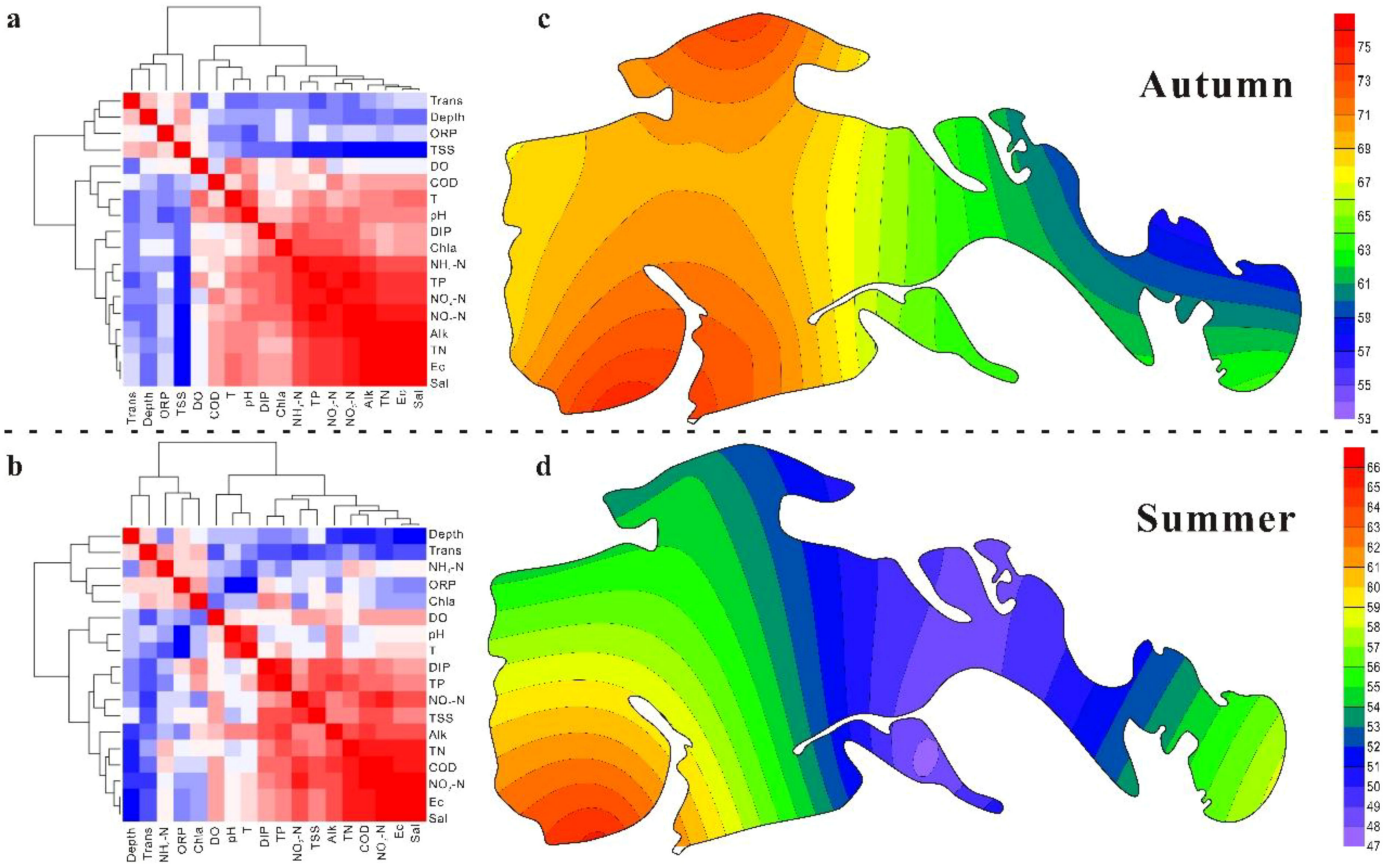
Correlation and cluster analysis of water chemistry **(a, b)** and the corresponding TLI in the wetland **(c, d)**. The dashed line is the boundary of the autumn (Upper parts) and summer (Lower parts).

Most water physicochemical parameters exhibited higher values in autumn than in summer (**Table 2**). Among them, DO, Trans, ORP, TSS, COD, Chl-a, TP, NH_4_-N, NO_3_-N, NO_2_-N, and DIP showed significant seasonal differences. Correlation and cluster analyses revealed that the measured variables could be divided into two major groups in both seasons (**Figure 4a, b**). The first group included Trans, Depth, and ORP, while the second group comprised Sal, Ec, Alk, TN, TP, COD, NO_3_-N, NO_2_-N, and DIP. These two groups were negatively correlated with one another. Within the second group, TN, TP, COD, NO_3_-N, NO_2_-N, and DIP were strongly positively correlated with Sal, Ec, and Alk. The principal component analysis further identified four principal components (**Supplementary Figures S2**, **S3**), explaining 83.45% and 84.30% of the total variance in autumn and summer, respectively. In autumn, the first component—dominated by pH, Ec, Sal, Alk, TSS, Chl-a, TN, TP, NH_4_-N, NO_3_-N, NO_2_-N, and DIP—accounted for 53.30% of the variance, far exceeding the combined 30.15% explained by the other three components. In summer, the first component (Ec, Sal, Alk, Depth, Trans, TSS, COD, TN, TP, NO_3_-N, and DIP) explained 42.30% of the variance. From the perspective of individual nutrient indicators, mean NO_3_-N concentrations corresponded to mesotrophic conditions, while TP levels were classified as eu-polytrophic in both seasons. In autumn, 50% of sampling sites were in a mesotrophic state based on NO_3_-N, whereas this proportion increased to 66.67% in summer. Conversely, TP showed higher levels of eutrophication, with 41.67% of sites classified as polytrophic in autumn and 83.33% as eu-polytrophic in summer. According to the TLI classification (**Supplementary Figure S3**; **Figures 4c, d**), the wetland was classified to middle eutropher and light eutropher in autumn and summer, respectively. Collectively, these findings indicate that the Ec, Sal, Alk, TSS, TN, TP, and NO_3_-N were the dominant factors controlling the water quality in the wetland.

**Table 2:** Ranges of water column variables in the study area.

Metrics	Autumn			Summer		
	Range	Mean	SD	Range	Mean	SD
T (°C)	19.0-24.2	21.71^a^	1.67	25.4-32.8	28.08^a^	2.58
pH	8.11-9.17	8.78^d^	0.30	7.80-9.43	8.76^d^	0.37
DO (mg L^-1^)	2.12-12.91	6.55^a^	2.43	1.21-1.73	1.49^a^	0.17
Ec (µs cm^-1^)	479.6-1888.0	1098.88^d^	558.77	380.2-2262	944.93^d^	523.52
Sal (ppt)	0.23-0.96	0.55^d^	0.29	0.18-1.14	0.46^d^	0.27
Alk (mmol L^-1^)	5.4-14.7	9.37^d^	3.52	4.0-19.0	8.79^d^	4.24
Depth (m)	0.6-1.5	1.11^c^	0.25	0.3-1.2	0.84^c^	0.25
Transparency (m)	0.2-1.3	0.72^b^	0.42	0.1-0.8	0.32^b^	0.20
ORP (mv)	46-119.5	78.28^a^	26.60	-154.3–107.23	-107.23^a^	26.12
TSS (mg L^-1^)	42.67-148.33	98.61^a^	33.87	10.67-48.67	23.08^a^	10.29
COD (mg L^-1^)	24.04-80.80	50.23^b^	19.99	0-63.28	25.17^b^	21.02
Chla (mg m^-3^)	8.217-67.914	27.24^a^	20.08	0-4.520	0.868^a^	1.377
TN (mg L^-1^)	0.473-2.039	1.074^d^	0.530	0.730-2.00	1.183^d^	0.362
TP (mg L^-1^)	0.036-0.195	0.096^a^	0.053	0.016-0.127	0.057^a^	0.036
NH_4_-N (mg L^-1^)	0.141-0.987	0.342^b^	0.241	0.019-0.145	0.058^b^	0.036
NO_3_-N (mg L^-1^)	0.170-0.836	0.411^b^	0.234	0.070-0.250	0.118^b^	0.056
NO_2_-N (mg L^-1^)	0.022-0.067	0.004^a^	0.001	0.010-0.020	0.019^a^	0.003
IP (mg L^-1^)	0.020-0.257	0.084^c^	0.080	0.050-0.062	0.028^c^	0.016

The significance levels represented by the letters a (p < 0.001), b (p < 0.01), c (p < 0.05), and d (p ≥ 0.05), based on the p-value.

### 3.2 Species assemblage and biomass distribution

**Figure 5:**
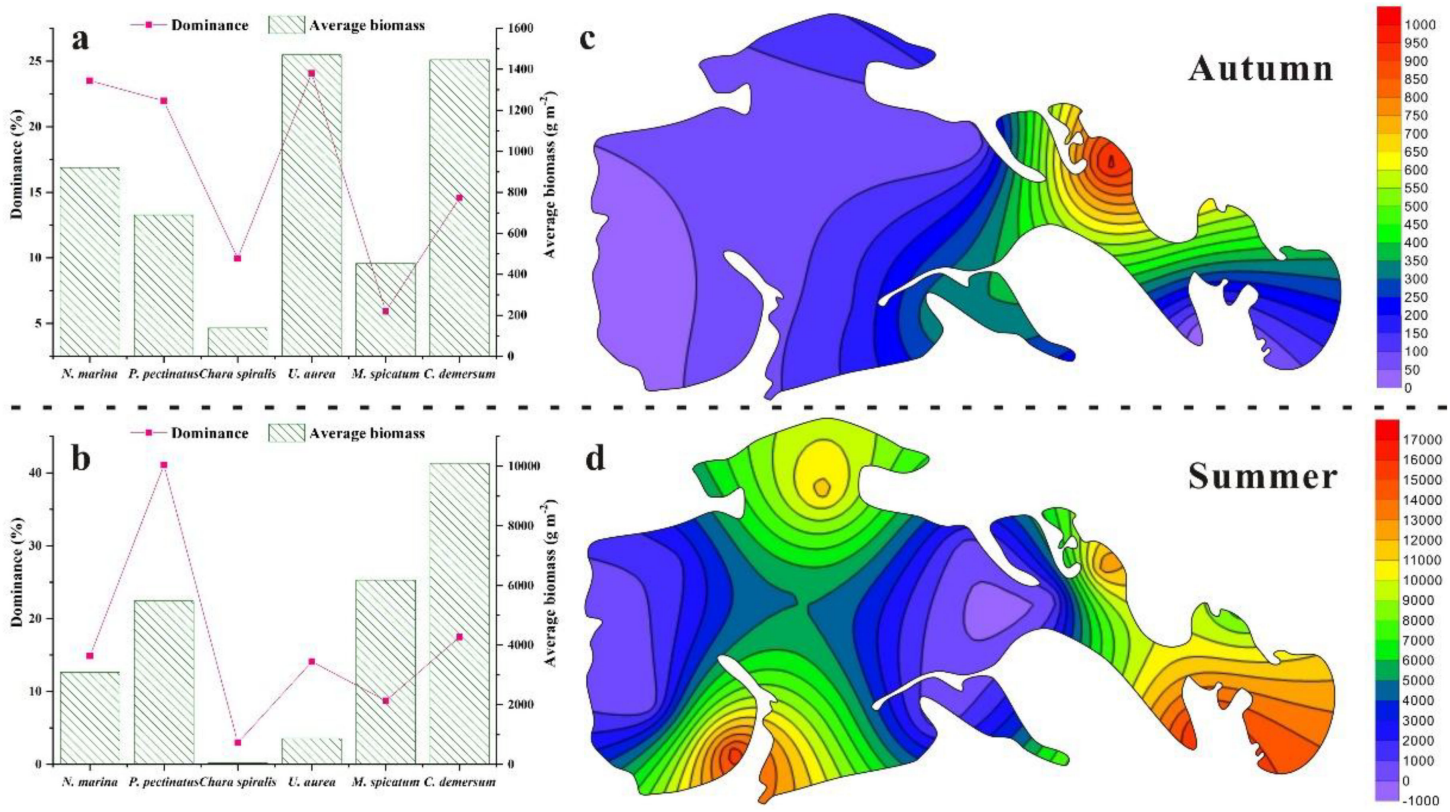
Species assemblage and biomass distribution in Autumn and Summer (The dashed line is their boundary). Figures with lowercase **(a, b)** represent the dominance and average biomass, and figures with the lowercase **(c, d)** represent the biomass distribution simulated from the average biomass in each sampling site.

Field surveys revealed that the species composition of submerged macrophytes remained relatively consistent across years, whereas species dominance and average biomass exhibited pronounced seasonal variations. Across the two survey periods, a total of eight species belonging to six families were recorded (**Table 3**). Among these, *N. marina*, *P. pectinatus*, *U. aurea*, *C. demersum*, *M.* sp*icatum*, and *Char.* sp*iralis* were consistently present in both surveys. In contrast, *P. distinctus* and *P. crispus* appeared only sporadically and were restricted to specific seasons. In terms of species dominance, *U. aurea* was the most dominant species in autumn, accounting for 24.07% of the total relative dominance, followed by *N. marina* (23.51%), *P. pectinatus* (21.97%), *C. demersum* (14.57%), *Char.* sp*iralis* (9.95%), and *M.* sp*icatum* (5.93%) (**Figure 5a**). In summer, the dominance hierarchy shifted, with *P. pectinatus* emerging as the leading species (41.07%), followed by *C. demersum* (17.49%), *N. marina* (14.89%), *U. aurea* (14.09%), *M.* sp*icatum* (8.72%), and *Char.* sp*iralis* (2.98%) (**Figure 5b**). Patterns of biomass distribution also varied substantially between seasons. In summer, *C. demersum* exhibited an overwhelming dominance, with a biomass of 10,100.01 g/m², whereas *Char.* sp*iralis* recorded the lowest biomass at 44.42 g/m². In autumn, *U. aurea* ranked first with 1,471.02 g/m², followed by *C. demersum* (1,446.75 g/m²), *N. marina* (920.28 g/m²), *P. pectinatus* (689.75 g/m²), *M.* sp*icatum* (453.13 g/m²), and *Char.* sp*iralis* (137.94 g/m²). Spatially, average biomass was highest in sheltered bay areas and lowest in open water zones, with a clear east-west gradient characterized by higher biomass in the eastern regions and lower biomass in the western regions (**Figures 5c, d**). This spatial pattern reflects the influence of hydrodynamic stability and environmental heterogeneity on submerged macrophyte growth and distribution.

**Table 3:** Classification of the recorded submerged macrophytes and values of the pH drift experiment.

Species	Families	Phenological period	pH drift experiment		
			Initial	Final	HCO_3_^-^ utilization
*Potamogeton pectinatus* L.	Potamogetonaceae	May - Oct	8.07	10.07 ± 0.02	Yes
*P. crispus* L.		Apr - Jul	8.07	10.05 ± 0.02	Yes
*P. distinctus* A. Benn.		May - Oct	–	–	Lack (Kadono, 1980)
*Najas marina* L.	Najadaceae	Aug - Oct	8.07	9.54 ± 0.08	Yes
*Utricularia aurea* Lour.	Lentibulariaceae	Jun - Dec	8.07	8.47 ± 0.34	Lack
*Ceratophyllum demersum* L.	Ceratophyllaceae	Jun - Oct	8.07	10.01 ± 0.01	Yes
*Myriophyllum* sp*icatum* L.	Haloragidaceae	Apr - Sept	8.07	10.22 ± 0	Yes
*Chara* sp*iralis*	Characeae	NA	–	–	Yes (Lucas et al., 1983)

“NA” means not available, “-” means no data available.

### 3.3 Relationship between hydrochemical properties and submerged macrophytes

**Figure 6:**
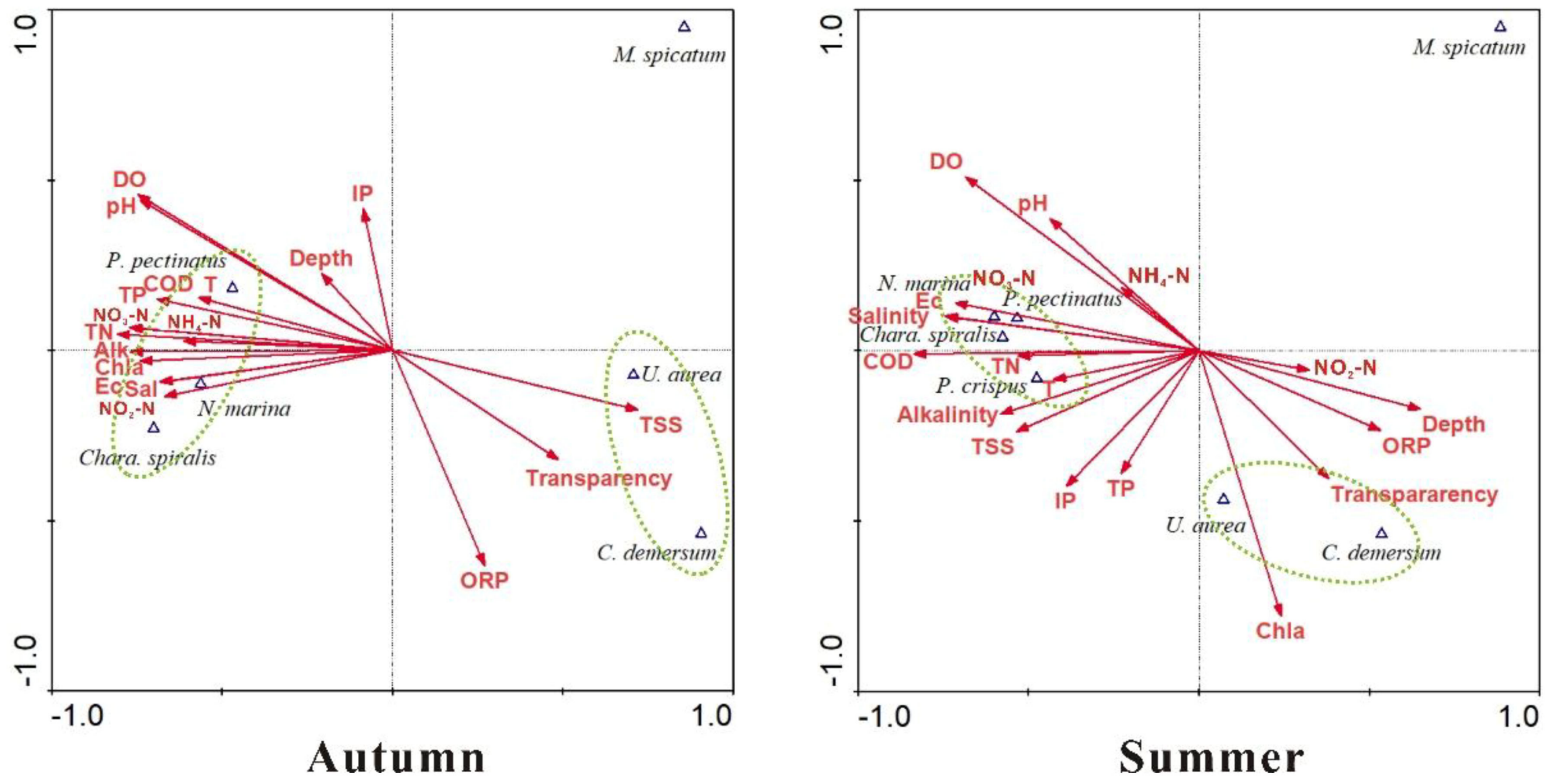
Canonical correspondence analysis between the water physicochemical variables and biomass distribution of the mutual species in both seasons. Submerged macrophytes circled in the same dashed ellipse are a group.

The pH-drift experiments (**Table 3**) revealed that the *M.* sp*icatum*, *C. demersum*, *P. pectinatus*, *P. crispus*, and *N. marina* are capable of utilizing HCO_3_^-^ as an alternative carbon source, whereas *U. aurea* lacks this ability. The canonical correspondence analysis (CCA) further demonstrated that submerged macrophytes could be classified into three distinct groups based on the relationships between their biomass distribution and water physicochemical properties (**Figure 6**). These groupings were highly consistent with the results of the cluster analysis of hydrochemical characteristics. The first group, consisting of *P. pectinatus*, *N. marina*, and *Char.* sp*iralis*—species present in both seasons—was strongly influenced by water column parameters such as T, pH, Ec, DO, Sal, Alk, COD, TN, TP, and NO_3_-N. The second group, represented by *U. aurea* and *C. demersum*, showed positive correlations with water Trans and ORP in both seasons, though with slight seasonal differences. In contrast, the third group, consisting solely of *M.* sp*icatum*, exhibited minimal correlation with hydrochemical parameters, indicating its broad adaptability to varying environmental conditions.

In summary, based on their adaptive capacity to water chemistry, submerged macrophytes in the study area can be ranked into three categories. *M.* sp*icatum* demonstrates the highest ecological resilience, thriving under a wide range of environmental conditions. The intermediate group includes *P. pectinatus*, *N. marina*, and *Char.* sp*iralis*, which have moderate tolerance and respond strongly to nutrient-related parameters. Finally, *U. aurea* and *C. demersum* are the most sensitive species, requiring higher water quality, particularly high transparency, to maintain growth and biomass stability.

## 4 Discussion

### 4.1 Factors governing water physicochemical properties

The geological background forms the foundation of the ecological environment and plays a vital role in understanding the formation and evolution of fundamental hydrochemical properties (Iversen et al., 2019; Zhang et al., 2023). This is particularly important in wetlands, where surface water and groundwater interact within a watershed, creating complex hydrochemical dynamics (Volik et al., 2023). At the global scale, the primary factor controlling solute chemistry is lithology, with natural waters strongly influenced by rock weathering within a given basin (Lakshmanan et al., 2003). Major ions, including K^+^, Na^+^, Ca²^+^, Mg²^+^, Cl^-^, SO_4_²^-^, HCO_3_^-^, and CO_3_²^-^, constitute a significant proportion of the total dissolved salts in wetland waters, and their concentrations largely depend on hydrogeochemical processes occurring in both surface and subsurface environments (Xiao et al., 2015). Consequently, analysis of major ion concentrations provides critical insights into their sources and governing processes. In this study, the γ(Na^+^ + K^+^)/γCl^-^ scatter diagram (**Figure 3b**) showed that nearly all sampling points were distributed above the 1:1 equilibrium line, indicating halite dissolution. Moreover, the relatively high sodium concentration compared to chloride suggests the contribution of silicate weathering. The abundance of Na^+^, Ca²^+^, and Mg²^+^ is associated with clay minerals such as montmorillonite, illite, and chlorite (Kumar et al., 2009). The γ(Ca²^+^ + Mg²^+^)/γ(HCO_3_^-^ + SO_4_²^-^) scatter diagram is particularly useful for distinguishing between carbonate and silicate weathering processes (Datta and Tyagi, 1996). In this study, both γ(Ca²^+^ + Mg²^+^)/γ(HCO_3_^-^ + SO_4_²^-^) and γ(SO_4_²^-^ + Mg²^+^)/γHCO_3_^-^ ratios (**Figure 3c, d**) were below the equilibrium line, indicating that silicate weathering, evaporite dissolution, and carbonate weathering are the dominant sources of major ions. Together, these processes are responsible for the elevated alkalinity and salinity characteristic of wetland waters (Wang et al., 2009; Lehmann et al., 2023). Over the past few decades, global climate change driven by intensive human activities has profoundly altered precipitation patterns and thermal regimes (Bandh et al., 2021), disrupting balances that had persisted over geological timescales, particularly in the Northern Hemisphere (Nosetto et al., 2008; Wang et al., 2003; Chen et al., 2018). Based on analyses of global datasets including rain, river, lake, and ocean water samples, Gibbs (1970) proposed three primary mechanisms controlling surface water chemistry: atmospheric precipitation, rock dominance, and evaporation–crystallization processes. In the present study, the Gibbs plot (**Figure 3a**) indicates that evaporation–crystallization is the dominant mechanism shaping water chemistry, consistent with the wetland waters are both saline and alkaline. The difference between precipitation and evaporation is one of the main drivers for the variations in water chemistry between seasons. Long-term climate records show a declining trend in annual precipitation and a simultaneous increase in atmospheric temperature over the past 30 years in the study area (Cui et al., 2016). These conditions have intensified the evaporation–crystallization process, further enhancing the fundamental water chemistry characteristics, particularly alkalinity and salinity. Thus, rock weathering combined with evaporation–crystallization processes constitute the primary control over water chemistry in this region.

Eutrophication, driven by excessive inputs of nitrogen and phosphorus, represents a pervasive and urgent global challenge to water quality (Barboza et al., 2019; Stevens, 2019). Classic studies of shallow lakes have shown that eutrophication drives ecosystem transitions between two alternative stable states: a clear-water state, characterized by high biodiversity and good water quality, and a turbid state, associated with low diversity and degraded ecological functions (Liu et al., 2020; Su et al., 2019). In this study, two distinct clusters of water quality were identified, corresponding to these states, suggesting that the studied wetland is currently undergoing a transitional phase from a clear to a turbid state. The primary drivers of this shift are anthropogenic activities, including excessive fertilizer use, industrial pollution, and wastewater discharge, which cause abrupt regime shifts (Folke et al., 2004; Wang et al., 2009). In the Momoge wetland, elevated concentrations of TN, TP, NO_3_-N, COD, TSS, NH_4_-N, NO_2_-N, and DIP are largely attributable to inputs from surrounding human activities, such as agriculture, industrial operations, and domestic activities in the watershed, particularly in the autumn, the drainage from the paddy directly flows into the Momoge wetland significantly increased the nutrient loading. This is one of the main reasons for the seasonal differences in nutrient states. In addition, the growing season of submerged macrophytes also contributes to the seasonal differences. Situated in the black soil region, the largest commercial grain production base in China (Cheng and Zhang, 2005), this area inevitably receives substantial nutrient runoff from agricultural practices and land-use change (Zou et al., 2018). For example, influent water samples showed TN and TP concentrations ranging from 0.92–9.85 mg L^-^¹ and 0.08–1.55 mg L^-^¹, respectively (Kou, 2012). Furthermore, 954 oil wells have been developed since 1986, with some extending into the core zone of the Momoge Nature Reserve (Wang et al., 2010), exacerbating contamination risks. In addition, wastewater from local residents directly contributes to water quality deterioration (Jiang et al., 2007). Beyond nutrient loading, external stressors such as wind-induced disturbance and the overexploitation of surface and groundwater also influence water quality by affecting transparency and water table fluctuations. This is supported by both cluster analysis (CA) and principal component analysis (PCA) results, which identify the TSS as one of the main controlling factors. Overall, at the regional scale, excessive nutrient inputs from anthropogenic sources, compounded by external physical disturbances, are driving a persistent and severe alteration of hydrochemical properties, causing the wetland to shift progressively from an oligotrophic to a eutrophic state.

### 4.2 Water physicochemical properties shape the distribution of submerged macrophytes

Submerged macrophytes interact closely with the aquatic environment in which they are anchored, and the dominance of particular species is largely a consequence of environmental selection (Liu et al., 2020). On the one hand, through photosynthesis, submerged macrophytes assimilate inorganic carbon, nutrients, and other dissolved minerals, thereby playing a crucial role in improving water quality and facilitating ecological restoration (Jeppesen et al., 2012). Conversely, the composition and distribution of submerged macrophyte communities are strongly shaped by the water environment, particularly the availability and form of dissolved inorganic carbon, which serves as the foundation for photosynthesis (Blowes et al., 2019). Unlike terrestrial plants, submerged macrophytes can utilize both CO_2_ and HCO_3_^-^ as carbon sources. Numerous studies have highlighted that species capable of utilizing HCO_3_^-^ exhibit greater adaptability to diverse aquatic environments (Maberly and Gontero, 2017; Wang et al., 2015; Yin et al., 2017). In this study, HCO_3_^-^ overwhelmingly dominated over CO_2_ in the water column, a direct outcome of the region’s geological background. Results from our pH-drift experiments indicate that most species possess the ability to utilize HCO_3_^-^. Specifically, *P. crispus*, *P. pectinatus*, *C. demersum*, *N. marina*, and *M.* sp*icatum* can use HCO_3_^-^ as an alternative carbon source, consistent with the findings of Yin et al. (2017). In contrast, *U. aurea* cannot utilize HCO_3_^-^, likely because it has evolved an alternative nutritional strategy—capturing zooplankton as a supplementary food source (Rutishauser, 1993). Although HCO_3_^-^ is generally less favorable for submerged macrophyte metabolism compared to CO_2_, the dominant species in this wetland have successfully adapted over long evolutionary timescales to thrive under these conditions. This indicates that alkaline water environments impose a strong environmental filter, favoring species capable of utilizing HCO_3_^-^ while excluding those unable to do so.

The biomass distribution of submerged macrophyte species is strongly regulated by the eutrophication level of the waters they inhabit (Dar et al., 2014; Dudgeon et al., 2006). Excessive nutrient loading, particularly N and P, profoundly disrupts aquatic ecosystems, leading to reduced biodiversity and shifts in dominant species (Ibáñez and Peñuelas, 2019). Our results demonstrate that the hydrochemical properties of the wetland can be classified into two distinct categories, and the biomass patterns of submerged macrophytes closely align with this classification. *P. pectinatus*, *N. marina*, and *Char.* sp*iralis* exhibited high biomass in nutrient-rich waters with elevated N and P concentrations, whereas *U. aurea* and *C. demersum* were primarily associated with clearer waters of higher transparency. This pattern suggests that as eutrophication intensifies due to human activities, the plant community will increasingly be dominated by species such as *P. pectinatus*, *N. marina*, and *Char.* sp*iralis*, which are well-adapted to low-transparency, nutrient-enriched environments (Tobiessen and Snow, 1984; Agami et al., 1980). This phenomenon may contradict with some conventional recognitions, partly because the wetland is undergoing the regime shift processes. In addition to nutrient effects, external disturbances play a significant role in shaping plant distribution. Global climate change and overexploitation of water resources have caused a 70% loss of open water area in the wetland (Chen et al., 2018, 2020), consistent with our findings of increasing water salinity. Submerged macrophytes are highly sensitive to water table fluctuations and wind-induced disturbances (Blowes et al., 2019). Long-term meteorological data indicate that the mean annual wind speed in this region is 3.7 m s^-^¹ (Cui et al., 2016), creating intense wave turbulence that threatens the stability and anchorage of submerged macrophyte communities (Sand-Jensen and Pedersen, 1999). Field observations further confirm that submerged macrophytes thrive primarily in sheltered zones, such as areas surrounded by *Phragmites australis* or *Scirpus planiculmis*, which act as natural barriers that buffer wave action and create relatively stable microhabitats. These protective zones reduce mechanical stress, allowing for the establishment and persistence of submerged vegetation. In general, the distribution and biomass of submerged macrophytes in this wetland are shaped by the synergistic effects of nutrient enrichment and external disturbances. Nutrient enrichment, primarily from human activities, drives shifts toward communities dominated by eutrophication-tolerant species. External disturbances, such as wind-driven turbulence and water level fluctuations, further influence spatial heterogeneity by favoring submerged macrophytes in sheltered microhabitats. Together, these factors dictate community structure, with water physicochemical properties acting as the central driver of submerged macrophyte dynamics.

### 4.3 Management applications

In recent decades, considerable efforts have been devoted worldwide to wetland restoration (Bertassello et al., 2025; Jiang et al., 2024). In the long term, the successful restoration of wetlands critically depends on the stable recovery of submerged macrophyte communities, which serve as the ecological foundation of these systems (Rybak et al., 2024). At the catchment scale, geological background determines the fundamental hydrochemical characteristics, exerting an initial environmental filter on submerged macrophyte species by providing different DIC forms (Iversen et al., 2019). At the local scale, massive nutrient inputs from anthropogenic activities, combined with external disturbances, create complex and overlapping effects on submerged macrophyte biomass and spatial distribution (Ibáñez and Peñuelas, 2019). Therefore, effective restoration strategies must integrate both geological context and trophic state to guide the rehabilitation of submerged vegetation in aquatic ecosystems. Although hydrological management approaches have been proposed to maintain water supply and ecological function in the Momoge wetland (Cui et al., 2016), our findings indicate that maintaining hydrological regimes alone is insufficient to prevent further degradation of this fragile ecosystem. This is especially true for submerged macrophyte communities, which function as cornerstone species in aquatic ecosystem restoration (Jeppesen et al., 2012). Here, we advocate a species configuration and pilot selection strategy as a pivotal approach for restoring submerged macrophytes in the Songnen Plain. Given the region’s alkaline water environment, submerged macrophytes should possess the ability to utilize HCO_3_^-^ for photosynthesis, as this trait enables them to colonize a wide range of habitats and persist under challenging conditions (Hussner et al., 2016; Maberly et al., 2015). The recovery process of submerged vegetation is gradual, involving a series of ecological shifts rather than a sudden reversal (Sand-Jensen et al., 2000; Sayer et al., 2010). Based on the relationships between submerged macrophytes and water column variables, species selection should align with the prevailing trophic status and water transparency. Pioneering species such as *P. pectinatus*, *N. marina*, and *Char.* sp*iralis* should be introduced first in regions with poor water quality and low transparency, where their tolerance to eutrophication allows them to establish initial submerged macrophyte communities. As water quality improves, indigenous dominant species such as *U. aurea* and *C. demersum* should be gradually introduced to stabilize and consolidate restoration outcomes. Finally, incorporating ornamental species like *M.* sp*icatum* into restored patches can enhance biodiversity and ecological stability, supporting long-term resilience. This aligns with the findings of Liu et al. (2020), who demonstrated that the presence of three or more submerged macrophyte species significantly improves water clarity in eutrophic lakes. In addition to species selection, external environmental pressures, particularly wind-induced turbulence and water table fluctuations, should be minimized during the initial stages of restoration (Dudgeon et al., 2006; Reid et al., 2019). Our field observations show that submerged macrophyte biomass is highest in sheltered bay areas and lowest in open water zones, suggesting that restoration areas should be located in relatively stagnant regions with stable water levels. To achieve these conditions, eco-engineering measures such as constructing buffer zones with native emergent aquatic plants (e.g., *Phragmites australis* and *Scirpus planiculmis*) are recommended (Pan et al., 2006). These vegetative barriers can attenuate wind and wave energy, creating a more stable microenvironment that facilitates the establishment and persistence of submerged macrophyte communities.

## 5 Conclusion

In the Songnen Plain wetlands, water salinity and alkalinity are primarily governed by geological processes, particularly rock weathering and evaporation–crystallization. These natural controls set the baseline hydrochemical conditions, while nutrient enrichment from anthropogenic activities and external disturbances, such as wind-induced turbulence and water level fluctuations, impose further modifications on the water column. This combination of natural and human-driven factors has led to profound changes in wetland ecological dynamics. A total of eight submerged macrophyte species representing six families were identified in this study. Among them, *M.* sp*icatum*, *C. demersum*, *P. pectinatus*, *P. crispus*, and *N. marina* possess the ability to utilize HCO_3_^-^, the predominant DIC form in this alkaline environment. This physiological trait provides these species with a competitive advantage, enabling them to persist and dominate under conditions where CO_2_ is limited. In contrast, *U. aurea*, which cannot utilize HCO_3_^-^, relies on alternative nutritional strategies, such as zooplankton capture, to survive. The biomass distribution patterns of submerged macrophytes are strongly shaped by water quality gradients. *P. pectinatus*, *N. marina*, and *Char.* sp*iralis* exhibit high biomass in nutrient-rich, low-transparency waters, reflecting their tolerance to eutrophication. Conversely, *U. aurea* and *C. demersum* show a strong preference for clearer waters with high transparency, indicating their sensitivity to environmental degradation. Thus, the inorganic carbon regime, nutrient loading, and external physical disturbances collectively act as environmental filters, determining both species assemblages and biomass distributions within submerged macrophyte communities.

From a restoration perspective, our findings emphasize that hydrological management alone is insufficient to reverse wetland degradation. We advocate a hybrid restoration strategy that integrates species configuration and pilot selection to accelerate the recovery of submerged vegetation and associated ecosystem functions. In heavily degraded areas with poor water quality, pioneering species such as *P. pectinatus*, *N. marina*, and *Char.* sp*iralis* should be introduced first to establish a foundational plant community. As water quality improves, sensitive indigenous species like *U. aurea* and *C. demersum* can be gradually incorporated to stabilize and diversify the community. Finally, ornamental and structurally complex species such as *M.* sp*icatum* should be added to enhance biodiversity, ecological resilience, and long-term stability. Moreover, external environmental pressures, particularly wind and wave disturbances, should be mitigated during the initial stages of restoration. Creating sheltered microhabitats by constructing vegetative barriers using native emergent plants, such as *Phragmites australis* and *Scirpus planiculmis*, will help buffer wave energy and promote the establishment of submerged macrophytes. In summary, the sustainable restoration of Songnen Plain wetlands requires a comprehensive approach that addresses both geological constraints and human-induced stressors. By integrating species-specific functional traits, nutrient management, and physical habitat modifications, it is possible to restore submerged macrophyte communities, enhance biodiversity, and rebuild the ecological integrity of these fragile wetland ecosystems.

## Data Availability

The raw data supporting the conclusions of this article will be made available by the authors, without undue reservation.
