# Supplementary figures and images for: Water physicochemical properties shape the distribution of submerged macrophytes: implications for wetland restoration in Songnen Plain
Source: Front Plant Sci. 2025 Dec 5;16:1716202. doi: 10.3389/fpls.2025.1716202 (PMC12715016; doi:10.3389/fpls.2025.1716202)

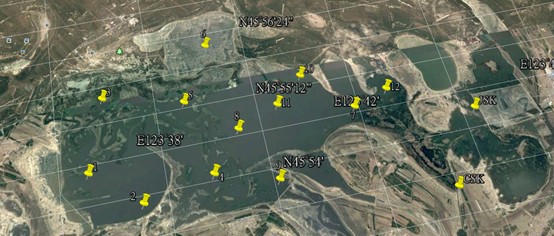

Supplement: Supplementary file 1 [file Image1.jpeg]

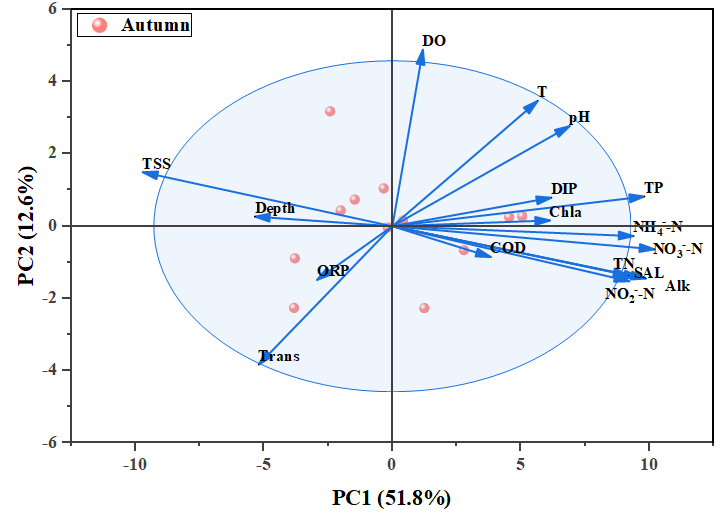

Supplement: Supplementary file 2 [file Image2.png]

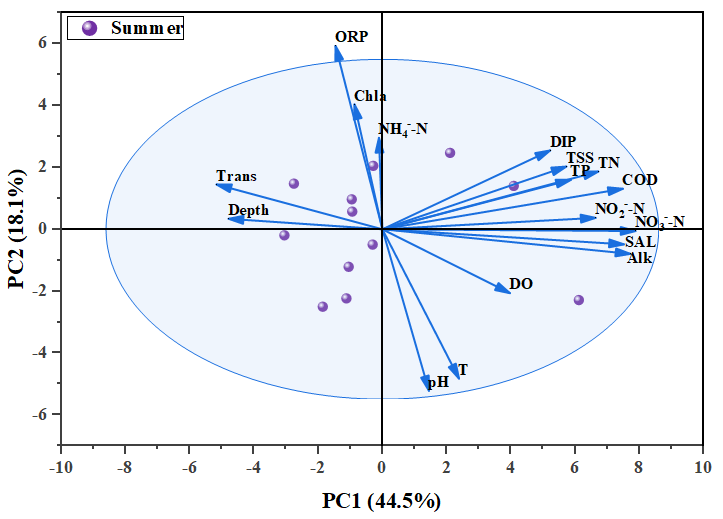

Supplement: Supplementary file 3 [file Image3.png]

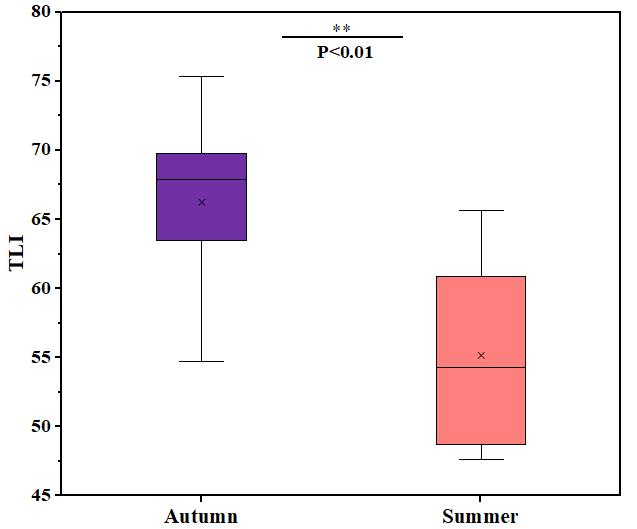

Supplement: Supplementary file 4 [file Image4.png]
